# Interplay Between CMGC Kinases Targeting SR Proteins and Viral Replication: Splicing and Beyond

**DOI:** 10.3389/fmicb.2021.658721

**Published:** 2021-03-29

**Authors:** Florentin Pastor, Lulzim Shkreta, Benoit Chabot, David Durantel, Anna Salvetti

**Affiliations:** ^1^International Center for Infectiology Research (CIRI), INSERM U1111, CNRS UMR5308, Université de Lyon (UCBL1), Lyon, France; ^2^Department of Microbiology and Infectious Diseases, Faculty of Medicine and Health Sciences, Université de Sherbrooke, Sherbrooke, QC, Canada

**Keywords:** CMGC kinases, serine/arginine-rich proteins, viral replication, RS domain, splicing

## Abstract

Protein phosphorylation constitutes a major post-translational modification that critically regulates the half-life, intra-cellular distribution, and activity of proteins. Among the large number of kinases that compose the human kinome tree, those targeting RNA-binding proteins, in particular serine/arginine-rich (SR) proteins, play a major role in the regulation of gene expression by controlling constitutive and alternative splicing. In humans, these kinases belong to the CMGC [Cyclin-dependent kinases (CDKs), Mitogen-activated protein kinases (MAPKs), Glycogen synthase kinases (GSKs), and Cdc2-like kinases (CLKs)] group and several studies indicate that they also control viral replication *via* direct or indirect mechanisms. The aim of this review is to describe known and emerging activities of CMGC kinases that share the common property to phosphorylate SR proteins, as well as their interplay with different families of viruses, in order to advance toward a comprehensive knowledge of their pro- or anti-viral phenotype and better assess possible translational opportunities.

## Introduction

**Figure 1:**
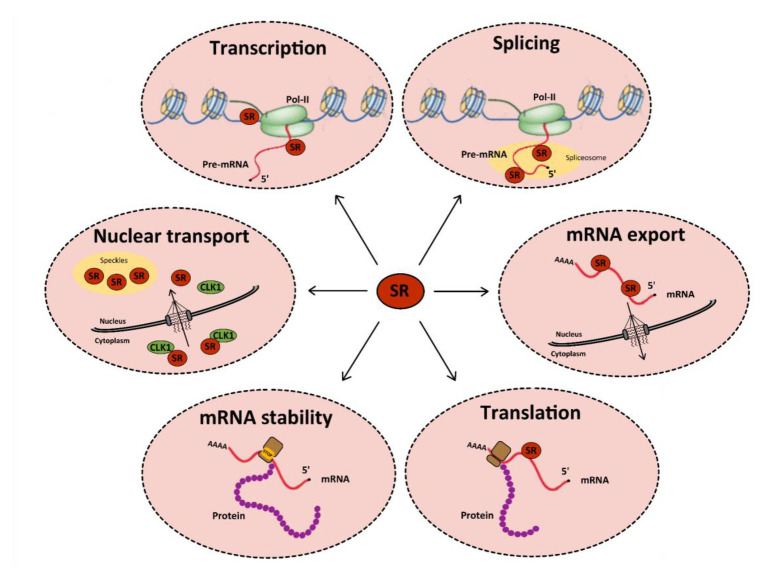
Functions of serine/arginine-rich (SR) proteins in mRNA metabolism. SR proteins (red circles) regulate transcription by activating the RNA polymerase II (in green). They also regulate constitutive and alternative splicing of pre-mRNAs by the spliceosome (in yellow). After splicing, SR proteins remain linked to mRNAs and some of them are involved in their nucleocytoplasmic export. In the cytoplasm, SR proteins can be redirected to the nucleus or regulate mRNA translation and/or stability: indeed, splicing regulation by SR proteins can induce the inclusion of a premature stop codon, which favors the recruitment of cellular proteins involved in RNA degradation by the non-sense mediated decay (NMD) pathway. Finally, some SR proteins can be involved in the nuclear import of Cdc2-like kinase 1 (CLK1). All these functions are tightly regulated by multi-site phosphorylation of SR proteins that also modulates their subcellular localization in the cytoplasm and the nucleus.

**Figure 2:**
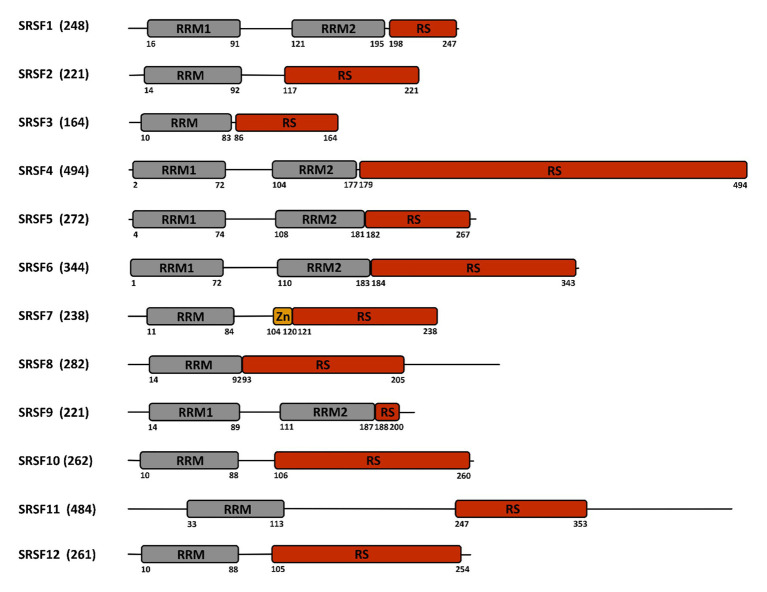
Structure of the SR-proteins family. The human family of SR proteins is composed by 12 proteins that share a similar modular organization with one or two RNA recognition motifs (RRM) in their N-terminal part, followed by a length-variable serine/arginine-rich (RS) domain in their C-terminal part. SRSF7 contains an additional zinc finger (Zn) domain. Numbers below the sequence refer to the amino acid position. The total number of amino acids is indicated between parentheses close to the protein name.

Cellular RNA binding proteins (RBPs) form a large and continuously expanding family of proteins that play fundamental roles at all steps of RNA metabolism, including transcription, splicing, transport, stability, and translation (Hentze et al., 2018). In addition, some RBPs also exert non-conventional activities on DNA by acting during DNA damage recognition and repair, mitosis, and regulation of telomere length (Naro et al., 2015). Among RBPs, serine/arginine-rich (SR) proteins constitute a conserved family of 12 proteins in humans that were initially discovered as factors required for constitutive and capable of regulating alternative splicing (AS; Long and Caceres, 2009; Howard and Sanford, 2015). In addition to their roles in initiating spliceosome assembly and helping define 5' and 3' splice sites, subsequent studies showed that besides their role in splicing, SR proteins, as other RBPs, have multiple additional functions in mRNA metabolism, including transcription, regulation of export of spliced mRNA from the nucleus, stabilization of cytoplasmic transcripts, and promotion of mRNA translation by ribosomes (Figure 1; see Anko, 2014 for a review). SR proteins share a common modular organization composed by one or more RNA-recognition motif (RRM) at their N-terminus, and a region (RS), rich in arginine-serine and/or serine-proline dipeptides at their C-terminus (Figure 2; Long and Caceres, 2009). The RRM domain interacts with exonic or intronic splicing regulatory elements. The RS domain can interact directly with RNA but is mostly involved in recruiting spliceosome components to splice sites (Shen and Green, 2006). Most SR proteins are mainly nuclear while some of them continuously shuttle between the nucleus and the cytoplasm (Twyffels et al., 2011). Within the nucleus, SR proteins are concentrated in nuclear speckles that were initially considered as storage sites for splicing factors (Spector and Lamond, 2011). Further studies indicated that several other factors involved in RNA metabolism also localized in speckles that are in proximity to transcriptionally active chromatin (Galganski et al., 2017). As other membrane-less nuclear bodies, speckles are formed by proteins, DNA and RNA components that condensate through a process of liquid-liquid phase separation and that can reversibly dissociate upon external stimuli or during the cell cycle (Strom and Brangwynne, 2019; Greig et al., 2020).

**Figure 3:**
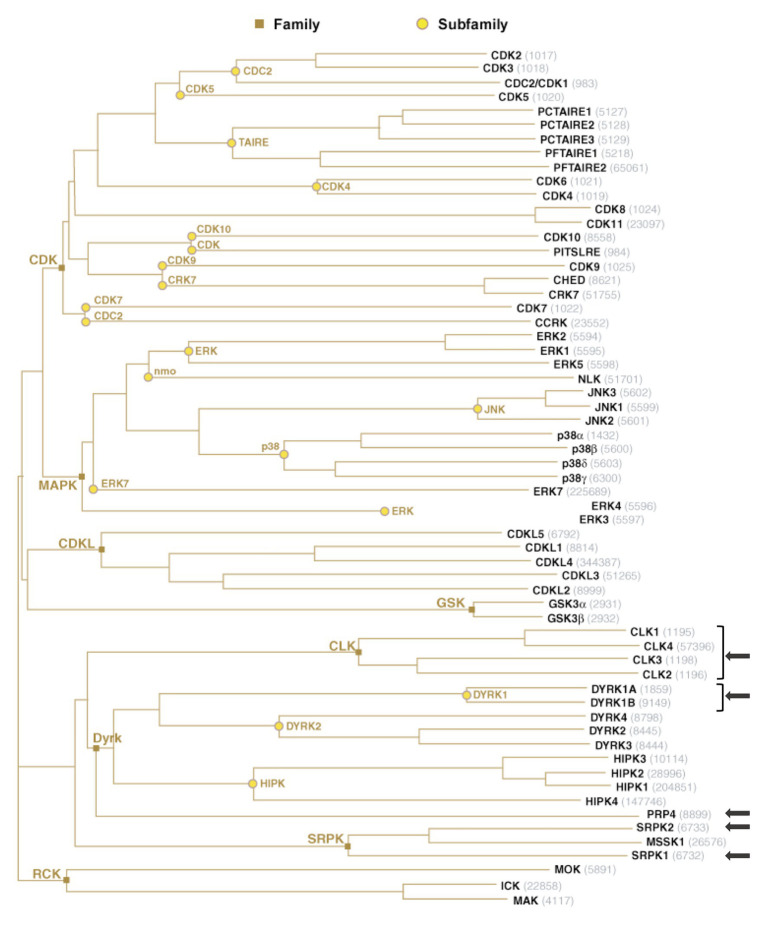
The group of human CMGC kinases. Of the 518 human protein kinases, 61 belong to the CMGC group and can be clustered into eight families and several sub-families of increasing sequence similarity and biochemical function. The unrooted kinase dendrogram shows the sequence similarity between the catalytic domains of the CMGC kinases: the distance along the branches between two kinases is proportional to the divergence between their sequences. Arrows indicate the kinases in SR proteins phosphorylation. NCBI accession number are indicated in gray. Illustration reproduced courtesy of Cell Signaling Technology, Inc. (www.cellsignal.com).

Importantly, reversible phosphorylation of serine residues within the RS region of SR proteins constitutes an essential mechanism that controls their intra-cellular and intra-nuclear localization, their affinity and specificity to RNA and their splicing activities (Caceres et al., 1997; Xiao and Manley, 1997; Misteli et al., 1998; Lai et al., 2000, 2001; Lin et al., 2005). Protein phosphorylation constitutes one of the most prevalent post-translational modifications that targets more than 30% of cellular proteins and controls a multitude of cellular processes, in particular those involved in signal transduction (Manning et al., 2002a). In humans, this reversible modification is performed by protein kinases that add a phosphate group to the side chain of one or several amino-acids, usually serine, threonine, or tyrosine, resulting in a change of hydrophobicity of the target protein that in turns modulates its conformation, localization, and interaction with other cellular proteins or with nucleic acids (Manning et al., 2002b; Patwardhan and Miller, 2007; Ubersax and Ferrell, 2007; Ardito et al., 2017). This phenomenon is constantly reversed by dephosphorylation catalyzed by phosphatases (Arena et al., 2005). Not surprisingly, the RS domain of SR proteins is also extensively modified by phosphorylation. Several kinases have been reported to phosphorylate SR proteins. All these kinases belong to the CMGC [Cyclin-dependent kinases (CDKs), Mitogen-activated protein kinases (MAPKs), Glycogen synthase kinases (GSKs), and Cdc2-like kinases (CLKs)] group that includes eight families of highly inter-connected kinases that regulate a variety of processes and, in particular, transcription and RNA processing (Varjosalo et al., 2013; Figure 3).

Not surprisingly, several studies evidenced interactions between these kinases and viruses. These interactions, that involve phosphorylation/dephosphorylation of cellular and/or viral targets, may result from the capacity of viruses to hijack these kinases to manipulate cellular pathways (modification of cellular and/or viral transcription, splicing, export of RNAs, …) in order to optimize their replication, and/or from a cellular response to viral infection. Interestingly, reports of interactions concern not only viruses that replicate and/or persist in the nucleus of the infected cell, but also viruses that have a cytoplasmic life cycle and, hence, a majority of RNA viruses. To illustrate the importance of these interactions, an increasing number of studies report the use of inhibitors targeting these kinases as potential anti-viral agents.

## Cellular Functions and Regulatory Mechanisms of CMGC Kinases Targeting SR Proteins

The main kinases reported to phosphorylate SR proteins are SR protein kinases (SRPKs; Gui et al., 1994a; Kuroyanagi et al., 1998; Wang et al., 1998) and CLKs (Ben-David et al., 1991; Howell et al., 1991; Johnson and Smith, 1991; Nayler et al., 1997; Duncan et al., 1998). However, several studies highlighted that these kinases also impact other activities unrelated to splicing. Conversely, some kinases, in particular dual-specificity tyrosine-regulated kinases (DYRKs; Alvarez et al., 2003; de Graaf et al., 2004; Shi et al., 2008; Toiber et al., 2010; Qian et al., 2011; Ding et al., 2012; Yin et al., 2012) and pre-mRNA processing 4 (PRP4; Alahari et al., 1993; Kojima et al., 2001), which were initially described for their function on other biological processes, appear to be equally able to phosphorylate SR proteins. This section describes the families of kinases targeting SR proteins, their mode of regulation, and their activities on SR proteins, as well as on other targets and/or processes not necessarily related to splicing. Because of their multiple activities, aberrant expression and/or activity of all these kinases have been frequently associated to cancer development. For studies regarding the role of these kinases in cancer, we refer the interested readers to dedicated reviews and references therein (Czubaty and Piekielko-Witkowska, 2017; Nikas et al., 2019; Laham et al., 2020).

### SR-Protein Kinases

The family of SRPKs includes three members, SRPK1, 2, and 3 that are produced from paralog genes with different tissue expression patterns. While SRPK1 is ubiquitously expressed, the two others are restricted to tissues such as brain, male germ cells (SRPK2), and muscle (SRPK3).^1^ SRPK1 was the first SR proteins kinase discovered, followed by SRPK2, and it remains the best studied (Gui et al., 1994a,b; Wang et al., 1998).

Their activity seems to be mainly controlled by their intra-cellular localization and interaction with other proteins. In interphase cells, SRPKs are mainly localized in the cytoplasm where they are found in complex with the Hsp70/90 chaperone complexes (Zhong et al., 2009; Zhou et al., 2012). The main signal releasing SRPKs from these complexes emanates from the EGF-AKT pathway that results in auto-phosphorylation of SRPK1 (Zhou et al., 2012). Osmotic stress or genotoxic agents are also signals promoting the dissociation of SRPK1 from cellular chaperones. This dissociation allows the phosphorylation of cytoplasmic SR proteins, their nuclear translocation as well as the nuclear import of SRPK1 itself. But SPRKs can also translocate in the nucleus in a cell cycle-dependent manner, in particular at the end of the G2 phase (Ding et al., 2006). In the nucleus, SRPK1 associates with SAFB, a component of the nuclear matrix that binds to scaffold/matrix attachment regions (SMARs) within DNA, to RNA, and several proteins to regulate nuclear activities such as transcription and splicing (Nikolakaki et al., 2001). Binding of SAFB to SRPK1 represses its kinase activity suggesting that this interaction is used to control SRPK1-dependent phosphorylation events within the nucleus (Tsianou et al., 2009).

**Figure 4:**
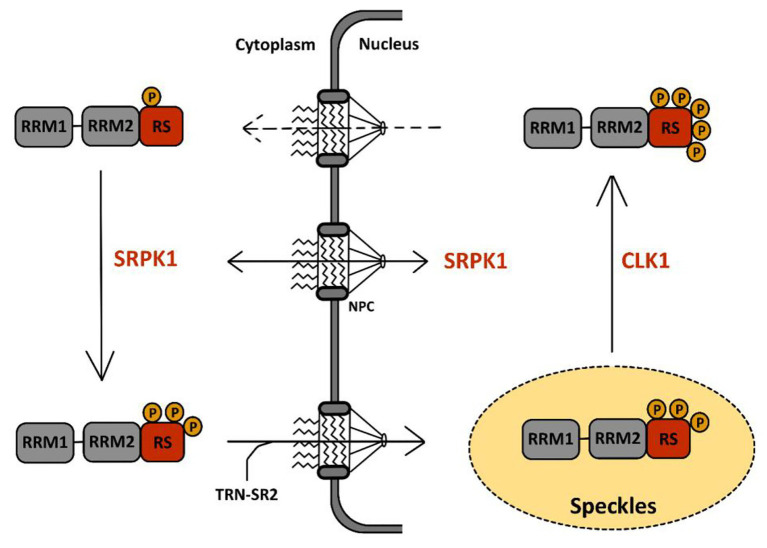
Phosphorylation cycle of SR proteins by serine-arginine protein kinase 1 (SRPK1) and CLK1. The sequence of events leading to the phosphorylation of SR proteins by SRPK1 and CLK1 has been well-documented for SRSF1, the prototype of the SR protein family, schematically represented here with two RRM domains followed by one RS domain. A first wave of SRSF1 phosphorylation takes place in the cytoplasm *via* SRPK1 that targets serine residues in its RS domain. This initial event triggers SRSF1 translocation to the nucleus through the nuclear pore complex (NPC) *via* association with the SR-specific transportin-2 (TRN-SR2) protein. Within the nucleus, SRSF1 accumulates in nuclear speckles (in yellow) together with other RNA-binding proteins. There, CLK1 further hyper-phosphorylates SRSF1 at serine residues in the RS domain. This second event drives the egress of SRSF1 from the speckles toward the nucleoplasm where it binds to pre-mRNA and to several other splicing factors to support co-transcriptional splicing. Dephosphorylation events by phosphatases are then required to complete the splicing reaction and for nuclear egress of SRSF1 together with spliced mRNA.

Serine-arginine protein kinases are highly specific for RS repeats and phosphorylate serine residues that are adjacent to arginine (Colwill et al., 1996a). The first described function of SRPKs concerns their effect on cellular splicing *via* phosphorylation of SR proteins. SRPKs are the kinases responsible for the first wave of phosphorylation of SR proteins in the cytoplasm after their synthesis as documented in the case of SRSF1 (Figure 4). This activity has been well-documented for SRPK1 and to a lesser extent for SRPK2 (Long et al., 2019). It leads to the nuclear import of SR proteins *via* the SR-specific transportin 2 (TRN-SR2) protein, a member of the β-karyopherin protein family (Lai et al., 2000, 2001). SRPKs also intervene later on in the nucleus where they assist CLKs (see below) during hyper-phosphorylation of SR proteins (Aubol et al., 2016).

Besides their direct effect on splicing, SRPKs also have additional roles potentially linked to their capacity to phosphorylate SR-like domains that are present in a variety of cellular proteins (Calarco et al., 2009). An example is provided by the reported interaction of SRPK1 with P1 protamine and Lamin B Receptor (LBR). SRPK1 phosphorylates LBR, a chromatin anchorage factor present on the inner surface of the nuclear membrane. Phosphorylation of LBR by SRPK1 occurs on its C-terminal SR-like domain at the onset of mitosis, following nuclear entry of SRPK1, and modulates its capacity to interact with chromatin (Mylonis et al., 2004; Sellis et al., 2012). SRPK1 also phosphorylates the P1 protamine in testis. Protamines are highly basic proteins that replace histones during spermatogenesis. Their association with DNA is highly dependent on phosphorylation events that also induce their transient association with LBR. The effect of SRPKs activity on the association of cellular factors with chromatin is also illustrated by studies showing that during the cell cycle, SRSF1 and SRSF3 display the capacity to bind chromatin in a phosphorylation-dependent manner. During interphase, both factors bind to chromatin by associating to histone H3 tail. At the onset of M phase, phosphorylation of SRSF1 and SRSF3 by SRPK1 contributes to their dissociation from H3 tails that are phosphorylated by Aurora B kinase. SRSF1 and SRSF3 subsequently re-associate with chromatin on post-mitotic chromosome when H3 phosphorylation decreases. It was suggested that this dynamic interaction may serve for cell cycle-dependent dissociation/association of other chromatin-associated factors, in particular, HP1 with whom both SRSF1 and SRSF3 interact (Loomis et al., 2009).

### Cdc2-Like Kinases

The CLK kinases represent the other most important family of kinases that control the activity of SR proteins. CLK1 is the first member of the family that was identified as a splicing kinase in a yeast two-hybrid screen (Colwill et al., 1996b). The CLKs family includes four members (CLK1–4) that share the EHLAMMERILGPLP motif, leading to the family name of LAMMER kinases (Colwill et al., 1996b). CLK1 and CLK4 are considered almost identical, whereas CLK3 is most distantly related.

Cdc2-like kinases are found mainly in the nucleus. Interestingly, the activity of human CLK1 can be regulated by alternative splicing of its RNA leading to skipping of exon 4 and production of a protein lacking kinase activity (Menegay et al., 2000; Uzor et al., 2018). Similarly, the murine CLK1/4 is regulated by a mechanism of intron retention in most murine tissues (Ninomiya et al., 2011). In both examples, production of an mRNA encoding an active kinase can be restored upon treatments that induce SR proteins de-phosphorylation and splicing arrest such as heat shock, osmotic stress, or the use of CLKs inhibitors (Ninomiya et al., 2011). These studies suggest that for both these kinases, control of alternative splicing of their pre-mRNA constitutes a mechanism to ensure rapid re-phosphorylation of splicing factors following a stress signal. Interestingly, a recent study indicated that the activity of CLK1/4 is temperature dependent with higher activity at lower temperatures correlating with a higher phosphorylation state of SR proteins and the regulation of AS of temperature-sensitive genes (Haltenhof et al., 2020). The abundance of CLK1/4 is also regulated during the cell cycle at the protein level by ubiquitin-mediated proteolysis. In particular, CLK1 levels peak during the G2/M phase to control the AS of a subset of cellular genes controlling cell cycle progression (Dominguez et al., 2016). Notably, such mechanisms (intron retention and cell-cycle dependent proteolysis) were not observed for CLK2, indicating a different mode of regulation.

Cdc2-like kinases are defined as dual-specificity kinases because they can phosphorylate both serine/threonine and tyrosine residues. However, while auto-phosphorylation occurs at tyrosine residues, phosphorylation of other substrates seems to target uniquely serines/threonines (Nayler et al., 1997; Menegay et al., 2000). Structural studies have shown that in contrast to SRPK1, CLK1 can phosphorylate serine residues flanked by either arginines (Arg-Ser) or prolines (Ser-Pro; Velazquez-Dones et al., 2005; Bullock et al., 2009). In addition, unlike SRPKs that possess a docking groove in their kinase domain for substrate recognition, CLKs use an N-terminal RS-rich domain, similar to that found in SR proteins, to bind to their substrate (Aubol et al., 2014; Keshwani et al., 2015). Most of their known functions are related to their capacity to phosphorylate several splicing factors, in particular SR proteins, and to regulate constitutive and alternative splicing. Recent studies have shown in detail how CLK1 participates together with SRPK1 in phosphorylation of SRSF1, the prototypical member of the SR family of proteins, illustrating both their difference in kinase activity as well as their cooperative interactions to phosphorylate SR proteins before their association to nascent RNAs (Figure 4). In particular, these studies have shown that upon phosphorylation by SRPK1 in the cytoplasm, SRSF1 is translocated into the nucleus and localizes within nuclear speckles. Phosphorylation by SRPK1 targets Arg-Ser dipeptides present in one of the RS domains (RS1) of SRSF1 from the C- to the N-terminus to generate a hypo-phosphorylated protein. Within speckles, SRSF1 is further hyper-phosphorylated by CLK1 within its RS2 domain, on Ser-Pro dipeptides. During this step, SRPK1 binds to CLK1, enhancing its kinase activity and also acting as a release factor for SRSF1 finally leading to the delocalization of this SR protein in the nucleoplasm and its engagement, together with other spliceosome components on nascent RNA (Aubol et al., 2016, 2018). Association between SRSF1 and CLK1 was shown to occur through the interaction between disordered domains present in the N-terminus of CLK1 and in the RS region of SRSF1. Interestingly, this interaction occurs in the cytoplasm and is involved in the nuclear import of CLK1 (George et al., 2019).

Despite extensive studies documenting how CLKs modulate phosphorylation of SR proteins and splicing, less information is available about their role on other cellular targets and pathways. Notably, CLK2 was shown to be required during the DNA damage response by associating with ATR and controlling its kinase activity (Collis et al., 2007; Rendtlew Danielsen et al., 2009). More recently, a study indicated that CLK1, 2, and 4 also localize to midbodies during cell division where they participate in the control of premature abscission of chromatin bridges during cytokinesis. This function was mediated by the CLK-dependent phosphorylation of Aurora B Kinase (Petsalaki and Zachos, 2016). Evidence for the involvement of CLKs during cell division is also provided by studies in trypanosomes that identified CLK1 and CLK2 as essential kinetochore components (Akiyoshi and Gull, 2014; Jones et al., 2014). Recently, a chemical compound that specifically inhibited CLK1 activity in trypanosomes was shown to impair formation of the kinetochore and lead to cell cycle arrest (Saldivia et al., 2020).

### DYRK1A/B

The DYRK subfamily of kinases is encoded by two groups of paralog genes (DYRK1A/B and DYRK2/3/4) that are conserved in all animal species. Their common feature is their function as priming kinases, in particular for glycogen synthase-3 (GSK-3) and Polo-like (PLK) kinases (Aranda et al., 2011).

In contrast to the other two main families of SR protein kinases, SRPKs and CLKs, DYRKs enzymes are mostly known for their activities in a wide variety of processes such as cell growth, differentiation and transcription but only a few studies have indicated that some of these enzymes, in particular DYRK1A, can regulate the activity of SR proteins (Aranda et al., 2011; Laham et al., 2020). DYRK1A and DYRK1B belong to the class I group of DYRKs kinases that are functionally different from those of class II group, which includes DYRK2, 3 and 4. The kinase was discovered in 1996 as highly expressed in the brain and its overexpression shown to be associated to some pathological traits of neurodegenerative syndromes, in particular Down syndrome (Guimera et al., 1996). Several studies further indicated a dosage dependent effect of DYRK1A on neuronal development with haploinsufficiency leading to intellectual disability, microcephaly, and autism whereas overexpression confirmed the association with clinical manifestations of trisomy 21 (Laham et al., 2020).

DYRK kinases are described as constitutively active kinases. Activation was thought to occur by co-translational auto-phosphorylation at tyrosine residues within their activation loop (Becker and Sippl, 2011). A recent study revised this notion by showing that autophosphorylation is primed by the hydroxylation of a proline residue by PHD1, an oxygen-dependent prolyl hydroxylase (Lee et al., 2020). Hydroxylation of DYRK1A and DYRK1B by PHD1 is required for the subsequent auto-phosphorylation and activation. Interestingly, the hydroxylated proline is located within a highly conserved region present in most kinases of the CMGC family suggesting that activation of these kinases by hydroxylation may constitute a common mechanism shared by all these kinases. Regulation of their enzymatic activity also occurs at the level of gene expression, notably by miRNA-mediated control of mRNA abundance (Chiu et al., 2019), and by interaction with regulating factors, in particular, with 14-3-3 proteins (Kim et al., 2004; Alvarez et al., 2007).

As all the members of this subfamily, DYRK1A is a dual-specificity kinase that can auto-phosphorylate on tyrosine and target its substrates on serine/threonine residues. DYRK1A and DYRK1B have a cytoplasmic and nuclear localization but, in the nucleus, only DYRK1A localizes to nuclear speckles and, as observed with CLK1, its overexpression induces speckle disassembly. A histidine-rich domain of DYRK1A constitutes the speckle targeting signal (Alvarez et al., 2003). Accordingly, DYRK1A phosphorylates the spliceosomal protein SF3b1, a component of the U2snRNP (de Graaf et al., 2006). In addition, several studies have reported that DYRK1A can phosphorylate SR proteins including SRSF1, SRSF2, and SRSF4 to 7 (de Graaf et al., 2004; Shi et al., 2008; Toiber et al., 2010; Qian et al., 2011; Ding et al., 2012; Yin et al., 2012). In particular, when overexpressed, DYRK1A can phosphorylate SRSF1, SRSF2, SRSF6 and SRSF7 and alter the alternative splicing of Tau, a neuronal microtubule-associated protein whose accumulation in neurofibrillary tangles in neurons is a hallmark of several neurodegenerative disorders and also observed in Down syndrome patients (Shi et al., 2008; Toiber et al., 2010; Qian et al., 2011; Ding et al., 2012; Yin et al., 2012). Similarly, phosphorylation of SRSF6 by DYRK1A alters splicing of cardiac troponin T (Lu and Yin, 2016).

As indicated above, DYRK1 kinases have pleiotropic activities on cellular pathways notably on cell proliferation and differentiation. Part of these activities seems to be mediated by the phosphorylation-dependent control of protein stability (Becker, 2012). In particular, both DYRK1A and 1B can induce the phosphorylation-dependent proteosomal degradation of Cyclin D1 to maintain cells in growth arrest. DYRK1A also controls the protein level of REST, a transcription factor that negatively regulates neuronal differentiation. These phosphorylation-dependent effects on protein stability may be due to the interaction of these kinases with components or adaptor proteins of ubiquitin ligases such as DCAF7 (Miyata and Nishida, 2011). But phosphorylation of selected proteins by both these kinases can also have the opposite effect and induce their stabilization as observed for CDK2 inhibitor p27^Kip1^ and for at least two other cellular targets of DYRK1A, the transcriptional factor NFATC1 and Preselinin 1, the component of the γ-secretase complex that plays an important role in generation of amyloid-β (Aβ; Ryu et al., 2010; Jung et al., 2011).

Transcription constitutes another important regulatory mechanism targeted by DYRK1A. In particular, DYRK1A is recruited to the proximal promoter of genes involved in cell growth and regulates their transcription by phosphorylating the C-terminal domain (CTD) of RNA polymerase II (RNA pol II; Di Vona et al., 2015). Interaction with the RNA pol II occurs *via* the association with DCAF that enables DYRK1A to interact with the polymerase CTD *via* its histidine-rich domain and to phosphorylate it (Lu et al., 2018; Yu et al., 2019). Regulation of transcription of selected genes by DYRK1A also occurs by interaction with p300/CBP (Li et al., 2018), to stimulate their histone acetyl transferase activity, and by phosphorylating histone H3 to modify its interaction with the transcriptional repressor HP1 (Jang et al., 2014).

Last, but not least, DYRK1A plays a role in DNA repair as suggested by a series of proteomic studies indicating that its nuclear interactome was enriched with factors involved in transcription and DNA repair. In particular, RNF169 was identified as a major DYRK1A interacting protein in three recent studies (Guard et al., 2019; Menon et al., 2019; Roewenstrunk et al., 2019). This protein is an E3 ubiquitin ligase involved in homologous DNA repair by counteracting the activity of 53BP1, a crucial non-homologous end-joining (NHEJ) driver. DYRK1A phosphorylates RNF169 at several sites and colocalizes with this DNA repair factor at damage foci. Phosphorylation of RNF169 by DYRK1A was required to limit 53BP1 deposition at H2A ubiquitin marks near the sites of damage. Accordingly, overexpression of active but not kinase-dead DYRK1A can reduce 53BP1 foci in a RNF169-dependent manner (Menon et al., 2019; Roewenstrunk et al., 2019). Interaction with RNF169 occurs also independently of DNA-damage and concerns both DYRK1A and DYRK1B, but not the class II DYRKs. Whether this interaction plays a role in cell’s survival after DNA damage or in other functions possibly unrelated to DNA repair still remains an open question. Importantly, DYRK1A can regulate cell proliferation and apoptosis by targeting caspase 9 during neuronal and retinal development (Laguna et al., 2008; Barallobre et al., 2014). DYRK1A can also promote cell survival after DNA damage by phosphorylating SIRT1, a NAD^+^-dependent protein deacetylase, thereby inducing the deacetylation of p53 (Guo et al., 2010).

### PRP4

The PRP4 kinase was initially identified in yeast as a temperature-sensitive factor involved in pre-mRNA processing and as a component of the U4/U6 snRNP (Banroques and Abelson, 1989; Rosenberg et al., 1991). Later on, the protein was characterized as a kinase able to phosphorylate SRSF1 *in vitro* and the human PRP4 gene cloned (Gross et al., 1997; Kojima et al., 2001). The PRP4 kinase displays some homologies with CLKs, in particular, a N-terminal RS domain. In interphase cells, the protein localizes in nuclear speckles and interacts with CLK1 that can phosphorylate the RS domain of PRP4 *in vitro* (Kojima et al., 2001; Dellaire et al., 2002). However, despite these initial strong homologies, no further evidence reported a role of PRP4 in SR proteins phosphorylation. Rather, the studies conducted in yeast and mammalian cells, suggest that this kinase is involved in spliceosome assembly by directly phosphorylating its components. In humans, PRP4 associates with the U5 snRNP and phosphorylates several components of the human spliceosomal B complex (Dellaire et al., 2002; Schneider et al., 2010). Interestingly, PRP4 is also a component of the N-CoR-2 deacetylase complex, suggesting that it coordinate splicing with transcription (Dellaire et al., 2002).

In addition to its role in splicing, several studies reported a function of PRP4 during cell division in both yeast and mammalian cells. While some of these effects may be due to the control of splicing of cell-cycle regulatory transcripts (Eckert et al., 2016), additional studies have shown that PRP4, as CLK1 and CLK2, can associate to mitotic chromosomes in yeast and humans, in particular to kinetochores during mitosis (Potashkin et al., 1998; Schwelnus et al., 2001; Montembault et al., 2007; Pozgajova et al., 2013; Islam et al., 2018). Additional reported activities also indicate a potential role in transcriptional regulation. Notably, in T lymphocytes PRP4 was reported to phosphorylate the Kruppel-like factor 13 (KLF13), a major transcription factor for CCL5 chemokine gene expression (Huang et al., 2007).

## Interplay with Viral Replication

This chapter describes currently reported interactions between DNA/RNA viruses and the kinases described above (Table 1). Studies on the role of SR proteins in viral replication are also mentioned.

**Table 1:** Interactions between viruses and kinases that target SR proteins: consequences on viral replication and cell’s functions.

Virus	Kinase(s)	Effect on the virus	Effect on the cell	References
HPV	SRPK1	Interacts with and phosphorylates E2 Interacts with E4	Increased SRPK1 expression and delocalization in the nucleus Downregulation of SRPK1 activity	Mole et al., 2020 Bell et al., 2007; Prescott et al., 2014
	DYRK1A/1B	Interacts and stabilizes E7 Interacts with E6	ND	Liang et al., 2008; Zhou et al., 2015 Kuroyanagi et al., 1998
HSV	SRPK1	Inhibition of viral splicing	Delocalizes SRPK1 in the nucleus and inhibits SRPK1-mediated SR proteins phosphorylation	Sciabica et al., 2003; Souki and Sandri-Goldin, 2009; Tunnicliffe et al., 2019
VZV	SRPK1	Interacts with and phosphorylates IE4	ND	Ote et al., 2009
CMV	SRPK1	ND	CMV infection increases SRPK1 levels in the cytoplasm	Gaddy et al., 2010
	DYRK1A/1B	Interaction with several immediate-early viral proteins	Increased expression of DYRK1A/1B and relocalization	Hamilton et al., 2018
EBV	SRPK2	Interacts with and phosphorylates BRLF2	ND	Duarte et al., 2013
AdV	CLK1,2 and 4	Regulates E1A premRNA alternative splicing	ND	Duncan et al., 1997; Yomoda et al., 2008
	DYRK1A/1B	Interacts with and phosphorylates E1A	Counteracts AdV transforming activity	Komorek et al., 2010; Cohen et al., 2013; Glenewinkel et al., 2016
HBV	SRPK1, SRPK2	Interacts with and phosphorylates Core. Promotes specific pgRNA packaging	ND	Daub et al., 2002; Zheng et al., 2005; Chen et al., 2011; Heger-Stevic et al., 2018
HIV	SRPK2	Increases viral production	ND	Fukuhara et al., 2006
	CLK1, CLK2	Control splicing of HIV RNAs and viral production	ND	Wong et al., 2011, 2013
	PRP4	Interacts with the N-terminus of Gag	Inhibition of SRSF1 phosphorylation	Bennett et al., 2004
	DYRK1A	Inhibits HIV-1 transcription	Phosphorylation and delocalization of NFAT Phosphorylation and degradation of Cyclin L2 Tat-mediated increase in DYRK1A and phospho-SC35 levels Dysregulation of Tau alternative splicing	Bol et al., 2011; Booiman et al., 2015; Kisaka et al., 2020 Kadri et al., 2015
IAV	CLK1	Impairs splicing of M1 RNA into M2 and viral propagation	ND	Karlas et al., 2010; Konig et al., 2010; Zu et al., 2015; Artarini et al., 2019
SindbisV	SRPK1	SRPK1 inhibitor SRPIN340 decreases viral replication	ND	Fukuhara et al., 2006
HCV	SRPK1	SRPK1 inhibitor SRPIN340 decreases viral replication	ND	Karakama et al., 2010
EBOV	SRPK1, SRPK2	Interacts with VP30 and phosphorylates it to modulate viral transcription	ND	Takamatsu et al., 2020
SARS-CoV	SRPK1	Interacts with, phosphorylates N protein, and modulates its multimerization.	ND	Peng et al., 2008

ND, not determined.

### Papillomaviruses

Human papillomaviruses (HPV) and in particular the high-risk HPV16 and 18 are the main causative agents of cervical neoplasia (Graham, 2017). HPVs are small non-enveloped viruses that have a double-stranded (ds) DNA genome that persists as a circular episome within the nucleus of host cells where viral replication and particles assembly also occur. HPV infects epithelial cells and its life cycle depends on the differentiation status of the cell, whereby low-level viral DNA amplification takes place in basal epithelial cells concomitantly with cell division. In contrast, a complete life cycle that includes expression of late viral genes leading to particle assembly can only occur in terminally differentiated keratinocytes (Graham, 2017). The balanced production of early and late viral proteins depends on several splicing events requiring the intervention of cellular splicing factors including SR proteins SRSF1, SRSF3, and SRSF9 (Cerasuolo et al., 2020). SRSF2 also contributes to the production of viral transcripts encoding the HPV16 E6 and E7 oncoproteins (McFarlane et al., 2015). Overall, SR proteins can make positive and negative contributions to the expression of a variety of viral HPV transcripts (Graham and Faizo, 2017). On the other hand, papillomavirus infection may in turn alter the expression and the phosphorylation of SR proteins, possibly to impact cellular precursor mRNA splicing. For example, the HPV16 E2 protein, an essential DNA-binding factor that regulates genome partitioning between daughter cells, viral replication and transcription, has been implicated in the upregulation of SRSF1, SRSF2, and SRSF3 (Klymenko et al., 2016; Mole et al., 2020). Notably, HPV proteins can interact with kinases involved in SR protein phosphorylation and in particular with SRPK1. The HPV E2 protein interacts with SRPK1, contributes to its accumulation, and is also a substrate of this kinase (Mole et al., 2020). Interestingly, the E2 protein of some HPV types has a serine/arginine-rich region that has been involved in the interaction with several cellular RNA-binding proteins including SR proteins (Graham, 2016). SRPK1 also interacts with E4, a viral factor that is highly expressed in differentiated keratinocytes and that has multiple functions during the late phases of the viral life cycle, notably *via* cell cycle arrest in G2-M phase and reorganization of the cytokeratin network (Graham, 2017). The protein is first synthetized as an E1-E4 fusion protein derived from a spliced E1-E4 transcript and multiple E4 isoforms are then produced by differential phosphorylation and proteolysis (Graham, 2017). E1-E4 interacts with and inactivates SRPK1, probably by sequestering it in cytoplasmic inclusion bodies resulting in hypo-phosphorylation of SR proteins and E2 as well (Bell et al., 2007; Prescott et al., 2014). Even if no data indicate that E2 can upregulate SRPK1, these studies suggest that E2 may be responsible for the SRPK1-mediated increase in SRSF1 phosphorylation that is observed in HPV-infected cells (Prescott et al., 2014; Mole et al., 2020). In contrast, E4 may counteract this effect by inhibiting SRPK1.

While evidence of interaction of HPV with other kinases such as CLKs is lacking, additional reports indicate that the DYRK1A/1B kinases can also interact with the HPV E6 and E7 late proteins, the two main viral oncogenes that are involved in cell cycle progression and inhibition of apoptosis. In particular, DYRK1A and DYRK1B phosphorylate E7 and, by doing so, increase its stability and potentially contribute to cell proliferation (Liang et al., 2008; Zhou et al., 2015). In contrast, interaction of E6 with DYRK1A was suggested to inversely correlate with the transformation potential of some HPV types (Kuppuswamy et al., 2013). Given that most of these studies were performed using transient expression models in which both the viral proteins and DYRK1A/B are overexpressed, it will be important in future studies to validate these interactions in more relevant physiological settings. Moreover, to address the role of SRPK, CLK, and DYRK1 kinases in HPV-associated tumor progression, it would be informative to compare the viral and cellular transcriptomes following the depletion or the pharmacological inhibition of each kinase.

### Herpes Viruses

Herpes viruses form a large family of enveloped viruses whose genome is composed of a double-stranded DNA molecule that encodes more than 90 viral factors. Herpes viruses can persist in infected cells in a latent form that can periodically reactivate to produce infectious particles. The large majority of herpes virus proteins are produced from unspliced RNAs. Therefore, herpes viruses have developed sophisticated strategies to ensure the efficient production of their viral proteins by counteracting cellular splicing and exploiting the cellular export machinery. The ICP27 protein of herpes simplex virus-1 (HSV-1) represents the prototypical protein of the herpes virus family that controls both these processes. ICP27 acts by inhibiting host-cell splicing and by hijacking the TAP/NXF1 export pathway to direct the nuclear export of intron-less viral transcripts (Hardy and Sandri-Goldin, 1994; Chen et al., 2002). ICP27 is expressed very early during the replicative phase and was the first viral protein described to interact with SRPK1 (Sciabica et al., 2003). This interaction leads to the delocalization of the kinase in the nucleus and to the hypo-phosphorylation of several SR proteins, resulting in splicing inhibition. Interaction with SRPK1 requires the ICP27 RGG box and inhibits the kinase by competing with its binding to endogenous SR proteins (Souki and Sandri-Goldin, 2009; Tunnicliffe et al., 2019). Even though most of HSV-1 proteins are produced from unspliced transcripts, splicing signals are found in many viral genes (Tang et al., 2019). The presence of such signals may play a role during the latent phase of viruses, when proteins like ICP27 are not present, to prevent accidental production of viral proteins. HSV-1 infection also promotes CLK2 expression in latently infected neurons (Kramer et al., 2003). Whether this expression is associated with a splicing function remains unclear. It is important to note that the impact of HSV-1 infection on cellular splicing and polyadenylation is likely not a complete inhibition. Indeed, changes in alternative polyadenylation and alternative splicing have been documented and some of these alterations may turn out as important to insure a productive infection and immune evasion (Hu et al., 2016; Wang et al., 2016; Tang et al., 2019; He et al., 2020).

Homologues of ICP27 displaying similar functions are found in all the other members of the herpes virus family. IE4, the Varicella-Zoster virus (VZV) homologue of ICP27, interacts with SRPK1 and is a target of its catalytic activity (Ote et al., 2009). While IE4 also interacts with SRSF1, SRSF3, and SRSF7, no impact of IE4 on SR protein phosphorylation or SRPK1 localization was described. Rather, it was suggested that IE4 phosphorylation by SRPK1 may promote its dissociation from viral RNA once it is exported to the cytoplasm (Ote et al., 2009). The EB2 protein of the Epstein-Barr virus (EBV), interacts with SRSF1, SRSF3, and SRSF7 and promotes the accumulation of viral mRNAs from intron-less genes by antagonizing the negative effect of SRSF3 (Juillard et al., 2012). SRSF3 is also recruited by the viral SM protein to alter cellular alternative splicing (Verma et al., 2010). Whether these processes are regulated by interaction with SR protein kinases is unknown. Interestingly, interaction with SRPK2 promotes the phosphorylation of BLRF2, a tegument protein possessing an RS motif, that is involved late steps of viral replication (Duarte et al., 2013).

In contrast to HSV-1, several Cytomegalovirus (CMV) proteins are translated from spliced transcripts (Gatherer et al., 2011; Balazs et al., 2017). CMV infection increases the abundance of SRPK1 in the cytoplasm of infected cells (Gaddy et al., 2010). Whether this modifies viral splicing or other steps of the CMV infectious cycle is presently unknown.

Recent studies indicate that herpes viruses can also interact with other kinases that target SR proteins. In particular, CMV infection upregulates expression of DYRK1A and DYRK1B. Compounds inhibiting these kinases can reduce viral replication at an early phase and similarly act on other herpes viruses such as HSV-1 and VZV (Hutterer et al., 2017). Accordingly, CMV infection of placental cells induces the upregulation of DYRK1A/DYRK1B expression at the mRNA and protein levels and delocalizes DYRK1A to cytoplasmic sites of virus assembly, and DYRK1B to nuclear viral replication compartments. Delocalization of these kinases was associated with their interaction with several viral proteins (Hamilton et al., 2018). The consequences of these interactions on splicing of cellular genes or on other steps of the viral life cycle were not determined.

### Adenoviruses

Adenoviruses (AdVs) constitute another large family of DNA viruses that replicate in the nucleus of the infected cells. In contrasts to herpes viruses, AdV extensively uses alternative splicing to regulate the expression of most of its genes (Zhao et al., 2014). SR protein phosphorylation plays a critical role in AdV pre-mRNA splicing. The viral E4-ORF4 protein binds to protein phosphatase 2A and interacts with the hyperphosphorylated form of SRSF1 and SRSF9 to activate their dephosphorylation which implements the splicing switch that occurs between early and late infection (Kanopka et al., 1998; Estmer Nilsson et al., 2001). Overexpressing SRSF1 blocks this splicing shift and is detrimental to viral replication (Molin and Akusjarvi, 2000). Studies on the effect of SR protein kinases have focused on the alternative splicing of the AdV E1A pre-mRNA. E1A is essential for AdV life cycle since it constitutes the major viral transactivator that also promotes entry of the cell into S-phase (Frisch and Mymryk, 2002). Its pre-mRNA is alternatively spliced to produce multiple proteins isoforms, two of which (13S and 12S) are predominant and carry out most of E1A’s functions. Using transient expression system, it was found that CLK1, 2, and 4 differentially regulate splicing of E1A mRNA to promote the production of a smaller 9S isoform (Duncan et al., 1997; Yomoda et al., 2008). Moreover, the CLK kinase inhibitor TG003 led to a rapid dephosphorylation of SRSF4, whose overexpression alters E1A splicing (Yomoda et al., 2008). However, neither of the studies investigated the effect of these modulations on viral replication nor examined the impact of these kinases on other AdV genes. Subsequent reports indicated that DYRK1A and DYRK1B interact with E1A *via* a C-terminal region that is conserved among all human AdVs. AdV mutants defective for the interaction with DYRK1A/1B were shown to be hyper-transforming as compared to wild type E1A, suggesting that interaction with these kinases may counteract its effects on the cell cycle (Komorek et al., 2010; Cohen et al., 2013). DCAF, the adaptor protein, that allows binding of DYRK1A to several cellular factors (Miyata and Nishida, 2011; Yu et al., 2019), also binds to E1A and mediates its association with DYRK1A and phosphorylation (Glenewinkel et al., 2016).

### Hepatitis B Virus

The Hepatitis B virus (HBV) genome persists in the nucleus of infected cells as a circular double-stranded DNA episome (cccDNA) that serves as a template for the transcription of viral RNAs (Seeger and Mason, 2015). The HBV RNAs required to produce all the viral constituents sufficient for production of infectious particles are unspliced. This observation suggests that, as described for herpes viruses, HBV may manipulate the host cell splicing and export machineries in order to express efficiently its viral genes. Nevertheless, HBV spliced RNAs are produced indicating that if such mechanism exists, it is not fully efficient (Sommer and Heise, 2008). The virus replicates in the cytoplasm *via* reverse-transcription of its pregenomic RNA (pgRNA) into a partially double-stranded and circular DNA molecule, a step that occurs after packaging of pgRNA into newly assembled capsids (Seeger and Mason, 2015). Initial studies indicated that phosphorylation of the HBV Core protein, the unique constituent of the capsid, is required in order to specifically package pgRNA associated to the viral polymerase (Selzer and Zlotnick, 2015). SRPK1 and SRPK2 bind and phosphorylate the HBV Core protein at several serine residues *in vitro*, suggesting that these kinases are required for pgRNA packaging (Daub et al., 2002). Interestingly, like other viral proteins that are phosphorylated by these kinases, the HBV Core protein contains a serine/arginine-rich domain resembling that found in SR proteins. Subsequent *in vitro* studies confirmed the interaction between Core and SRPK1/2 and also indicated that specific packaging of pgRNA by the Core proteins required a balanced phosphorylation/dephosphorylation of some of its serine residues (Zheng et al., 2005; Chen et al., 2011; Heger-Stevic et al., 2018). Despite these *in vitro* observations, the role of SRPK1 and SRPK2 in the phosphorylation of Core in infected cells is still a matter of debate. Also, the impact of SRPK1/2 on viral RNA splicing has not been investigated.

Besides being involved in nucleocapsid formation in the cytoplasm, the HBV Core protein is also abundantly present in the nucleus of infected hepatocytes where it may regulate viral gene expression (Akiba et al., 1987; Zhang et al., 2016). A recent proteomic analysis indicated that the nuclear interactome of Core was mainly composed by cellular RBPs and in particular by SR proteins, one of which, SRSF10 displays an antiviral activity (Chabrolles et al., 2020). Unexpectedly, however, depleting SRSF10 or provoking its dephosphorylation does not affect HBV RNA splicing but rather modulates the level of nascent HBV RNA. This study suggests that a kinase involved in SR proteins phosphorylation may additionally controls the HBV life cycle at an intra-nuclear step by targeting SRSF10 and potentially other SR proteins associated to Core. Such kinase(s) may also target the RS-like domain of Core to modulate its capacity to bind to viral DNA and/or RNA.

### Human Immunodeficiency Virus

Expression of the HIV proteins is finely tuned by alternative splicing of its RNAs produced from an integrated viral genome (Sertznig et al., 2018). Accordingly, SR proteins (including SRSF1, SRSF2, SRSF3, SRSF5, SRSF6, SRSF7, and SRSF10) and all their kinases, modulate HIV-1 pre-mRNA splicing, transcription, and replication (Paz et al., 2014; Mahiet and Swanson, 2016; Brillen et al., 2017; Shkreta et al., 2017). In particular, overexpression of SRPK2 increased HIV replication by stabilizing SR proteins levels in transfected cells. Surprisingly, however, no anti-viral effect was reported using SRPIN340, a specific inhibitor of SRPK1 and 2 (Fukuhara et al., 2006).

Overexpression of CLK1 and CLK2, respectively, enhanced HIV Gag protein production and inhibited HIV replication, while chlorhexidine, an inhibitor of CLK2, 3, and 4, altered HIV pre-mRNA splicing and blocked virus production (Wong et al., 2011, 2013). However, no interaction has been reported so far between these kinases and viral constituents. In contrast, HIV-1 and HIV-2 Gag proteins, the structural components of the viral nucleocapsid, interact with PRP4 *in vitro* and *in vivo* and inhibit the phosphorylation of SRSF1 (Bennett et al., 2004).

Finally, several reports indicate that the HIV life cycle is regulated by DYRK1A in macrophages. In these cells, that are considered a viral reservoir, HIV-1 replication was associated with a polymorphism within the 5'-UTR of DYRK1A (Bol et al., 2011). Even if the consequences of this polymorphism on the enzyme were not investigated, subsequent studies showed that DYRK1A can downregulate HIV-1 transcription likely by phosphorylating NFAT, a transcription factor of the HIV-1 LTR, and by inducing its translocation into the cytoplasm (Booiman et al., 2015). Further studies also pointed to a role of DYRK1A in the inhibition of HIV-1 replication in macrophages by phosphorylating Cyclin L2 and inducing its degradation (Kisaka et al., 2020). The antiviral effect of DYRK1A was shown to occur at the transcriptional level. Interestingly, Cyclin L2, like Cyclin L1, has an RS-like domain, localizes in speckles and is believed to be involved in splicing, suggesting that its proviral activities may also involve processing of HIV RNAs (Dickinson et al., 2002; de Graaf et al., 2004; Herrmann et al., 2007). Finally, DYRK1A was overexpressed in brain autopsies from HIV-positive patients with encephalitis. Upregulation of DYRK1A was also observed *in vitro*, in cells expressing the HIV Tat protein (Kadri et al., 2015). Overexpression of DYRK1A was associated with increased phosphorylation of SRSF2 (SC35) and dysregulated alternative splicing of Tau pre-mRNA, confirming the role of this kinase in the production of Tau splice variants associated with Tat-mediated neuropathies.

### Influenza Virus

Influenza virus (IAV) stands apart among the family of RNA viruses since it belongs to a small group of viruses that replicate in the nucleus despite the lack of a DNA intermediate (Dou et al., 2018). The viral genome is composed by eight negative sense-RNA segments, two of which, M and NS, encode transcripts that need to be spliced by hijacking the cellular machinery as shown by the relocalization of several splicing components upon viral infection (Dubois et al., 2014). Several SR proteins (e.g., SRSF2, SRSF3, and SRSF5) are important for viral replication, suggesting that their phosphorylation by CLK and/or SRPK is critical for viral protein production (Artarini et al., 2019). The SR protein SRSF10, which is phosphorylated by CLK and SRPK kinases (Shi and Manley, 2007), controls the alternative splicing of ANP32A, a cellular protein essential for replication of avian IAV in mammalian cells (Long et al., 2016), to produce splice variants that differentially impact viral replication (Fang et al., 2020). An initial genome-wide siRNA screen aimed at identifying factors important for IAV pointed to CLK1 as required for efficient viral replication. Consistently, treatment of cells with TG003, an inhibitor of CLK1, strongly reduced virus propagation by decreasing the level of spliced M2 viral RNA (Karlas et al., 2010; Konig et al., 2010). Further studies confirmed the involvement of CLK1 on IAV replication as well as the anti-viral effect of newly developed compounds targeting this enzyme. Notably, among other splicing kinases only SRPK1 and SRPK2 emerged as able to control viral production even though at a much lower level as compared to CLK1 (Artarini et al., 2019).

### Cytosolic Viruses: Hepatitis C Virus, Ebola Virus, Sindbis Virus, and Coronavirus

Most RNA viruses replicate in the cytoplasm where the viral polymerase is in charge of generating new genome molecules and viral mRNAs. Not surprisingly, fewer studies have explored their interaction with splicing kinases. However, even if not strictly located in the nucleus, most if not all RNA viruses also encode viral proteins that can transit into the nucleus and eventually hijack some of its components. One such example is the reovirus T1L, which encodes a protein that alters the subnuclear localization of SRSF2, which would in turn impact cellular pre-mRNA splicing to ultimately enhance reovirus replication (Rivera-Serrano et al., 2017).

On the other hand, SR proteins shuttle between the nucleus and the cytoplasm and have cytoplasmic functions (Twyffels et al., 2011). Likewise, most SR protein kinases are not strictly located in the nucleus and, as indicated above, also have additional activities besides regulating splicing. Accordingly, Fukuhara et al. (2006) showed that SRPIN340, an inhibitor of SRPK1 and 2 can inhibit propagation of Sindbis Virus. Further studies showed that SRPIN340 could also restrain replication of Hepatitis C virus (HCV) whereas SRPK1 overexpression increased it (Karakama et al., 2010). SRPK1 was also found to be an important regulator of Ebola virus (EBOV) transcription. This kinase is recruited within EBOV inclusion bodies where it interacts with and phosphorylates the viral transactivator VP30 (Takamatsu et al., 2020). Both inhibition of SRPK1 or its overexpression reduced viral replication, confirming the need for a balanced and tightly controlled phosphorylation of VP30 for EBOV transcription. SRPK1 can also phosphorylate the nucleocapsid protein (N) of Severe acute respiratory syndrome coronavirus (SARS-CoV). The main function of N is to package viral RNA during virion assembly. This protein has a central arginine/serine-rich domain, localized between an N-terminal RNA-binding and a C-terminal dimerization domain, that is highly conserved among different CoV strains. Previous studies reported that the N protein of SARS-CoV is phosphorylated by SRPK1 and GSK-3, another member of the CMGC family (Peng et al., 2008; Wu et al., 2009). In particular, phosphorylation of N by SRPK1 alters its capacity to multimerize but not its RNA binding activity (Peng et al., 2008). More recently, phospho-proteomic analyses confirmed that the N protein of SARS-CoV-2 is similarly phosphorylated in particular within the arginine/serine-rich region (Bouhaddou et al., 2020; Yaron et al., 2020). While infection with SARS-CoV-2 activates a set of kinases involved in cytoskeleton signaling and cell cycle regulation (Bouhaddou et al., 2020), a recent study showed that SRPK1/2 can phosphorylate N of SARS-CoV-2 *in vitro*. As for the N protein of SARS-CoV, phosphorylation by SRPK1/2 is thought to prime further phosphorylation events by GSK-3. Importantly, genetic invalidation or chemical inhibition of SRPK1/2 reduced SARS-CoV-2 replication in susceptible cell line as well as in primary human pneumocytes (Yaron et al., 2020).

## Conclusion and Perspectives

**Figure 5:**
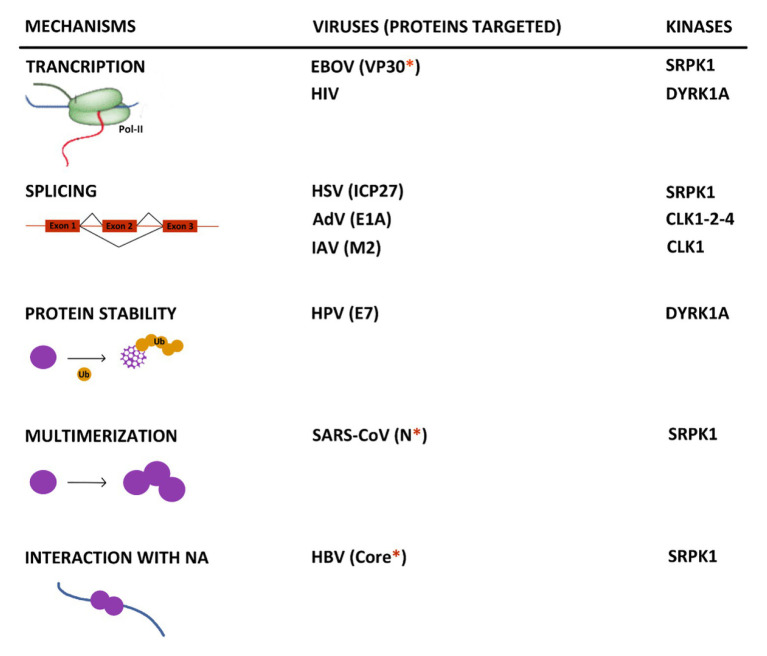
Summary of the major known effects resulting from the interplay between kinases that target SR proteins and viruses. When identified, the viral target of the kinase is indicated between brackets. The red asterisk indicates if the protein contains an arginine/serine-rich domain. NA, nucleic acid.

The increasing number of reports indicating an interplay between viruses and kinases involved in SR protein phosphorylation allows to highlight some important points. First, even if in most cases the mechanisms, underlying the pro- or antiviral activity of these kinases, were not uncovered, available data indicate that they exert their effects at multiple steps of viral life cycles (Figure 5). SRPK1 and DYRK1A can control the transcription of EBOV and HIV RNAs by phosphorylating viral and cellular factors (Booiman et al., 2015; Kisaka et al., 2020; Takamatsu et al., 2020). In the case of DYRK1A, this activity may also extend to other viruses thanks to its intrinsic capacity to phosphorylate the host RNA polymerase II CTD and modulate its activity. As expected, some viruses interact with these kinases to control the splicing of their viral RNAs as observed for HSV, AdV, HIV, and IAV (Duncan et al., 1997; Sciabica et al., 2003; Yomoda et al., 2008; Souki and Sandri-Goldin, 2009; Karlas et al., 2010; Wong et al., 2011, 2013; Artarini et al., 2019; Tunnicliffe et al., 2019). As shown for some cellular and viral factors, phosphorylation by some of these kinases may also regulate some intrinsic properties of their target such as protein stability (Liang et al., 2008), their capacity to multimerize (Peng et al., 2008) as well as to interact with nucleic acids (Heger-Stevic et al., 2018). Even if many of these observations need to be confirmed in more physiologically relevant infectious settings, they do suggest that hijacking of these kinase activities is critical for viral gene expression. Analyses of the virus-induced phospho-proteome may represent an interesting strategy to gain a larger view of the interaction between viruses and these kinases.

Second, it is striking to observe that many viral targets of these kinases contain a serine-arginine-rich domain that resembles that found in canonical SR proteins and several other cellular proteins (Calarco et al., 2009). This is the case for the HPV E2, the HBV Core, the EBOV VP30, the SARS-CoV2 N, and EBV BRLF2 proteins. It is tempting to speculate that the presence of such a domain may constitute a signature common to viral proteins regulated by this spectrum of kinases that could be used to develop targeted antiviral compounds.

Thirdly, very few studies have explored the impact of the interaction between these kinases and viral constituents on cell metabolism, in particular on the splicing landscape of the host. Indeed, several studies have documented that viral infections, caused by DNA or RNA viruses, can modulate splicing of cellular RNAs (Boudreault et al., 2019; Chauhan et al., 2019). Notably, a recent study on IAV indicates that the set of genes regulated by splicing following infection is different from those regulated at the transcriptional level (Thompson et al., 2020). While some of these splicing modulations may arise as a consequence of host response to infection, others may be due to a virus-directed effect on splicing kinases, in particular those targeting SR proteins. Future studies should focus on understanding how viral infections modulate the activity of these kinases and on the impact of these alterations on the cell physiology and fate. In addition, future studies should also evaluate whether kinase inhibitors could be envisaged to regulate viral replication and be used as therapeutic agents.

## Author Contributions

FP and AS planned the review. FP, LS, and AS wrote the paper. BC and DD gave suggestions and revised the manuscript. All authors contributed to the article and approved the submitted version.

### Conflict of Interest

The authors declare that the research was conducted in the absence of any commercial or financial relationships that could be construed as a potential conflict of interest.

## Footnotes

^1^www.proteinatlas.org
